# Immune Status in Children Before Liver Transplantation—A Cross-Sectional Analysis Within the ChilsSFree Multicentre Cohort Study

**DOI:** 10.3389/fimmu.2019.00052

**Published:** 2019-01-25

**Authors:** Tamara Möhring, André Karch, Christine S. Falk, Tobias Laue, Lorenzo D'Antiga, Dominique Debray, Loreto Hierro, Deirdre Kelly, Valerie McLin, Patrick McKiernan, Joanna Pawlowska, Piotr Czubkowski, Rafael T. Mikolajczyk, Ulrich Baumann, Imeke Goldschmidt

**Affiliations:** ^1^Research Group Epidemiological and Statistical Methods (ESME), Helmholtz Centre for Infection Research, Braunschweig, Germany; ^2^Division of Pediatric Gastroenterology and Hepatology, Department of Paediatric Liver, Kidney and Metabolic Diseases, Hannover Medical School, Hannover, Germany; ^3^European Paediatric Liver Transplantation Network EPLTN, Hannover, Germany; ^4^German Center for Infection Research, TTU-IICH Hannover, Braunschweig, Germany; ^5^Institute for Epidemiology and Social Medicine, University of Münster, Münster, Germany; ^6^Institute of Transplant Immunology, Hannover Medical School, Hannover, Germany; ^7^Ospedali Riuniti di Bergamo, Bergamo, Italy; ^8^Hôpital Necker-Enfants Malades, Paris, France; ^9^Hospital Infantil Universitario La Paz, Madrid, Spain; ^10^Birmingham Children's Hospital, Birmingham, United Kingdom; ^11^Service Spécialités Pédiatriques, Genève, Switzerland; ^12^Centre for Rare Diseases Therapy, Children's Hospital of Pittsburgh, Pittsburgh, PN, United States; ^13^Department of Gastroenterology, Hepatology, Nutritional Disorders and Pediatrics, The Children's Memorial Health Institute, Warsaw, Poland; ^14^Institute of Medical Epidemiology, Biostatistics and Medical Informatics, University of Halle, Halle, Germany

**Keywords:** pediatric, liver transplantation, immune monitoring, cytokines, pre-transplant, hepatology, lymphocyte subsets

## Abstract

**Background:** Both, markers of cellular immunity and serum cytokines have been proposed as potential biomarkers for graft rejection *after* liver transplantation. However, no good prognostic model is available for the prediction of acute cellular rejection. The impact of underlying disease and demographic factors on immune status *before* pediatric liver transplantation (pLTx) is still poorly understood. We investigated expression of immune markers before pLTx, in order to better understand the pre-transplant immune status. Improved knowledge of the impact of pre-transplant variables may enhance our understanding of immunological changes post pLTx in the future.

**Methods:** This is a cross-sectional analysis of data from the ChilSFree study, a European multicentre cohort study investigating the longitudinal patterns of immune response before and after pLTx. Immune cell counts and soluble immune markers were measured in 155 children 1–30 days before pLTx by TruCount analysis and BioPlex assays. Results were logarithmised due to skewed distributions and then compared according to age, sex, and diagnosis using *t*-tests, ANOVAs, and Tukey *post-hoc* tests. The association between immune markers at time of pLTx and patients' age was assessed using a fractional polynomial approach. Multivariable regression models were used to assess the relative contribution of each factor.

**Results:** Sex had no effect on immune status. We found strong evidence for age-specific differences in the immune status. The majority of immune markers decreased in a log-linear way with increasing age. T and B cells showed a sharp increase within the first months of life followed by a log-linear decline in older age groups. Several immune markers were strongly associated with underlying diagnoses. The effects of age and underlying disease remained virtually unchanged when adjusting for each other in multivariable models.

**Discussion:** We show for the first time that age and diagnosis are major independent determinants of cellular and soluble immune marker levels in children with end-stage liver disease. These results need to be considered for future research on predictive immune monitoring after pLTx.

## Introduction

Immune monitoring after liver transplantation (LTx) has been proposed as an approach for predicting the occurrence of immunological and infectious complications (1, 2). It might, thereby, offer a tool to guide dosing of immunosuppressive therapy. The role of the immune system, however, in the pathogenesis of the primary underlying liver disease and in the process of recovery after LTx is yet not fully understood. Although it has been shown that markers of cellular immunity differ by age in healthy children (3) and although some diseases leading to LTx are associated with an endogenous or exogenous immunosuppression (4), it is still unknown if and to which extent demographic factors and disease characteristics affect markers of cellular immunity and their associated soluble factors in children with severe liver disease. If the immunological set-up differs before transplantation, it must be assumed that these differences will also impact the immune response after transplantation. Findings on immune monitoring after LTx might, therefore, need to take into account pre-transplant variables, and might not be easily generalizable without this information. Our study aims at the investigation of variables that influence immune status before pediatric LTx, thereby, identifying factors that need to be incorporated into analyses for the prediction of immunological and infectious complications after LTx. To this end, we use data from a prospective European multicentre cohort study of liver transplanted children and assess the functional form of association between sex, age, and underlying disease on the one hand and immune monitoring markers on the other hand at the time of liver transplantation.

## Materials and Methods

### Study Design

Our analysis uses cross-sectional data from the prospective international multicentre cohort study ChilSFree (5). ChilSFree is conducted by a consortium of seven European pediatric liver transplant centers that joined the EnprEMA-recognized European Pediatric Liver Transplant Network EPLTN. The study focuses on assessing the effect of post-transplant immunosuppression and immune reconstitution patterns on the risk of graft rejection and infectious complications after pediatric liver transplantation. The study protocol comprises one visit before liver transplantation and seven visits in the first year after liver transplantation. At each visit, clinical data is collected and added to an electronic case reporting form (eCRF) in a Marvin-administered database (xclinical, Munich, Germany). In addition, whole blood samples are collected according to predefined protocols and are shipped per express post to the centralized immunological laboratory in Hanover, Germany, where the immunological analyses are conducted.

Eligible for inclusion into ChilSFree are patients with an age below 18, who undergo *de novo* liver transplantation. Exclusion criteria are history of a previous liver transplantation, and any underlying conditions that may interfere with the patient's safety, compliance or study evaluation in the opinion of the local investigator. All enrolled patients receive immunosuppressive therapy according to specific local protocols. For the current analysis only data from the pre-LTx visit were used.

### Leukocyte Subset Analyses and Quantification of Immune Mediators

Whole blood samples were collected in a sterile EDTA tube, stored at room temperature (20–25°C) and shipped per express post (maximum of 2 days) to the Institute of Transplant Immunology. All samples were immediately processed for flow cytometry; EDTA plasma was stored at −80°C until measurement of cytokine and chemokine levels by multiplex array analyses. Lymphocyte subsets were measured by BD Trucount^TM^ tubes and standardized flow cytometry (LSR II, Becton Dickinson, USA). Monoclonal antibody mixtures and 50 μl EDTA blood were added to the BD Trucount^TM^ tube according to manufacturer's instructions. In each tube, the defined number of fluorescent beads allowed the calculation of absolute numbers (cells/μl) of each cell type based on a fixed formula. Soluble immune markers were determined using a Luminex-based multiplex approach and 50 μl EDTA plasma according to the manufacturer's instructions (Bio-Rad, Hercules, USA). Two panels (human cytokine panels 1 and 2, angiogenesis panel) with a total number of 49 different cytokines, chemokines, and tissue-associated factors were analyzed and at least 50 beads per analyte per sample or standard were recorded and the analysis was performed with the BioPlx Manager 6.1.1 software an 5-parameter logistic plots (Table 1). In preparation for the study, the effect of shipment on cell numbers and cytokine/chemokine levels was investigated by sending samples via standard mail to our own lab; moreover, samples were exposed to storage at 4°c vs. room temperature (23°C) for 24 and 48 h to explore the effect of temperature. In addition, different anticoagulance tubes (EDTA, Na-Heparinat, Li-Heparinat and Citrate) were used since anticoagulation is known to be an important factor for the measurement of soluble immune factors. EDTA blood provided the most stable conditions for a period of 48 h so that it was used throughout the entire study. In TruCount FACS data, stable T, B, NK cell, monocyte, and even neutrophil cell counts were found in EDTA blood shipped or stored for 24 h or 48 h with <1% cell loss.

**Table 1:** Soluble immune markers analyzed in the ChilSFree study.

**Cytokines**		**Synonym**	**Chemokines**		**Synonym**
TH1 responses	IFN-γ		CCL chemokines	CCL2	MCP-1
	IL-2			CCL3	MIP-1α
	IL-12(p70)			CCL4	MIP-1β
	G-SCF			CCL5	RANTES
	GM-CSF			CCL7	MCP-3
	TNF-α			CCL11	Eotaxin
TH2 responses	IL-4			CCL27	CTACK
	IL-5		CXCL chemokines	CXCL1	Gro-a
	IL-10			CXCL8	IL-8
	IL-13			CXCL9	MIG
TH9 responses	IL-9			CXCL10	IP-10
TH17 responses	IL-17			CXCL12	SDF-1α
	IL-23 (IL12p40)		**Growth factors**	M-CSF	
Polyfunctional	IL-1α			SCF	
	IL-1β			SCGF	
	IL-1RA			PDGF	
	IL-3			HGF	
	IL-6			FGF–β	
	IL-7			MIF	
	IL16			TNF-β	LT–α
	IL16				
	IL-18				
	IFN-α2				
	LIF				
**Angiogenic factor**	VEGF		**Soluble surface molecules**	sCD25	IL-2Rα
				ICAM-1	
				VCAM	
				TRAIL	

### Statistical Analyses

To achieve the objectives of this study, we first assessed the association of the three potential influencing factors (sex, age, primary diagnosis) with each immune monitoring parameters individually before building multivariable models to quantify the relative effect of each influencing factor when controlled for the other two. For this, immune monitoring parameters were assessed for their distribution, and were logarithmised when necessary. The association of immune monitoring parameters with sex was assessed by *t*-tests or Wilcoxon rank-sum tests (where appropriate). The effect of age was investigated using linear regression models allowing for non-linear effects based on the fractional polynomial approach as described by Royston et al. (6). With this approach it was assessed if the use of a polynomial or a combination of two polynomials fitted the underlying data better than a linear term only. By using the Stata 14 MFP environment for the fractional polynomial approach, a closed test procedure was applied to find the best fitting fractional polynomial model (linear, one polynomial term or two polynomial terms) to avoid overfitting. Details about the closed test procedure can be found in (6–11) and the Stata MFP help file. The association of age and immune monitoring parameters was visualized using a scatter plot together with the fractional polynomial regression fit and corresponding 95% confidence limits. Underlying diagnosis was categorized in four disease groups (biliary atresia, acute liver failure, tumor, others) based on pathophysiological considerations to minimize within-group heterogeneity in this study population with a variety of primary diagnosis. The association between diagnosis and immune monitoring parameters was investigated using one-way ANOVAs for global testing; in case of evidence for differences between groups, Tukey *post-hoc* tests were used for pairwise comparisons (adjusted for multiple testing). Box plots of logarithmised parameter values (boxes represent the 25, 50, and 75% percentile; whiskers are defined as the upper/lower box boundary plus/minus 1.5 times the interquartile range) were used for visualization. In order to assess the effects of sex, age, and underlying disease when controlled for each other multivariable models were built using a multivariable fractional polynomial approach as provided by the MFP command in Stata 14 (7–9). Here, the effect of continuous variables (in this study age) is modeled in the same way as described above for the univariable analysis but adjusted for the other variables in the model. The effect of transplant center was assessed by adding a random effect to the final multivariable models. The stability of the multivariable fractional polynomial models was investigated using the bootstrapping approach as implemented in the MFPBOOT package in Stata 14 (10, 11). With this approach bootstrapped samples (*n* = 1.000) of the original study population were exposed to the same multivariable fractional polynomial model building approach to check if the selected fractional polynomial models were stable toward small changes in the composition of the study population. All analyses were performed on an exploratory basis only; *p*-values were, thus, not adjusted for multiple testing.

### Ethics Approval

Ethics approval was obtained from the Institutional Ethics Committee of Hannover Medical School, as well as from all local Ethics Committees in the seven centers. All study participants and/or their legal guardians provided written informed consent.

## Results

### Study Population

Of the 254 patients enrolled in the ChilSFree study, 151 patients with data available for both cellular and soluble immune markers could be included in this cross-sectional analysis (Table 2). Median age of study participants was 2.8 years (interquartile range: 1.0–9.1 years). The most common diagnosis was biliary atresia (31.8%).

**Table 2:** Baseline characteristics of study participants (*n* = 151).

**Characteristics**	**n (%)/Median (interquartile range)**
**Sex**	
Boys	80 (52.9%)
Girls	71 (47.1%)
**Age at transplantation**	2.8 (1.0–9.1)
0–2 years	42.7%
3–10 years	36.6%
11–18 years	20.7%
**Underlying diagnosis**	
Biliary atresia	49 (32.5%)
Hepatoblastoma	13 (8.6%)
Acute liver failure	8 (5.3%)
Metabolic liver disease	17 (11.3%)
Alagille syndrome	7 (4.6%)
Cystic fibrosis	7 (4.6%)
PFIC	11 (7.3%)
Sclerosing cholangitis	7 (4.6%)
Morbus Wilson	4 (2.7%)
Toxic liver damage	1 (0.7%)
Autoimmune hepatitis	0
Congenital hepatic fibrosis	0
Other	27 (17.9)

### Effect of Sex on Pre-LTx Immune Markers

We found no systematic effect of sex on immune cells (Supplementary Table 1). While interleukin (IL) IL-5 (*p* = 0.034) and IL-6 (*p* = 0.030) values were significantly lower in girls than in boys, this is consistent with the number of expected chance findings due to multiple testing. All other cytokine and chemokine levels did not show sex-specific differences.

### Effect of Age on Pre-LTx Immune Markers

**Figure 1:**
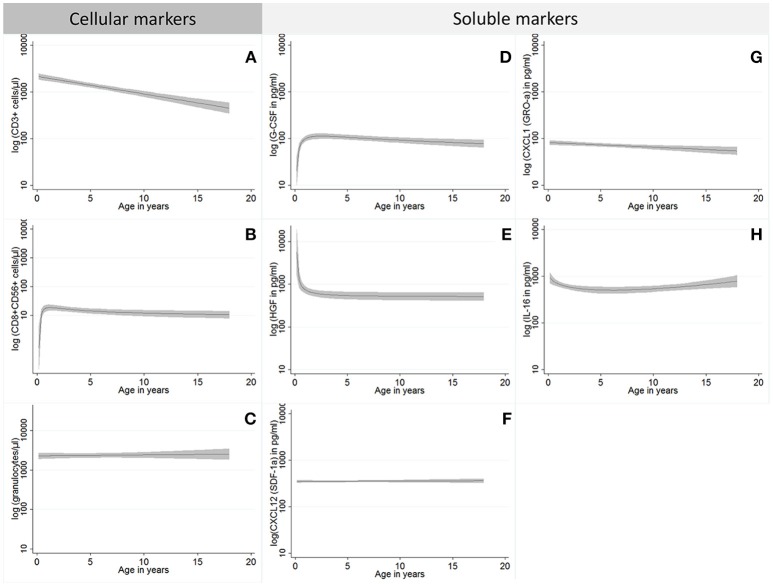
Overview of the functional forms for the association between immune parameters and patients' age; the functional forms identified in this study for cellular **(A–C)** and soluble markers **(D–H)** are visualized in this figure using one proxy example from each group. Functional forms identified for cellular markers comprise log linear decreases without (**A**; all cell types except for those described in **B,C**) and with a dip at zero (**B**; CD8^+^CD56^+^ T cells), and the group without age effect (**C**; granulocytes). For soluble markers we differentiate functional forms with log linear decreases without [**G**; GM-CSF, CXCL1 (GRO- α), IL12-p70, IL-17, IL2-Rα (sCD25), IL-9, CXCL10 (IP-10), M-CSF, CCL4 (MIP-1 β)] and with a dip at zero [D; ÍL-2, CCL11 (Eotaxin), IL-1β, FGF-b, G-CSF, IFN-γ, IL-13, IL-5, IL-4, IL-17, CCL5 (RANTES), and TNF-α], those with a more extreme than a log linear decrease [**E**; CXCL8 (IL-8), SCGF-b, and HGF], those with a decrease followed by an increase (H; IL-16 and VCAM1) and the group without age effect [**F**; CCL27 (CTACK), ICAM1, IFN-α2, IL-10, IL-12p40, IL-15, IL-18, IL-1α, IL-1RA, IL-3, IL-6, LIF, CCL2 (MCP1), CCL7 (MCP-3), MIF, CXCL9 (MIG), CCL3 (MIP-1α, PDGF-bb, SCF, CXCL12 (SDF-1α), TNF-β, TRAIL, and VEGF]. Displayed are fractional polynomial regression functions (black line) with corresponding confidence bands (gray).

Age was strongly associated with the majority of the investigated immune markers before LTx. This includes all immune cell numbers which displayed a log linear decrease with increasing age (Figure 1A), with granulocytes as the only exception (Figure 1C). In addition, one cellular immune subset, i.e., CD8^+^CD56^+^ T cells, showed a dip at zero in addition to the log linear decrease (Figure 1B). Since T cell development is completely thymus-dependent, the observed log linear decrease may be mainly associated with the declining thymic output during this early phase in the development of the T cell repertoire. It also affected B, NK cells, and monocytes and, hence, may represent hallmarks of early leukocyte development also in children with end-stage liver disease. In healthy newborns, the kinetics of these immune cells, especially CD8^+^CD56^+^ T cells have not been investigated in detail yet; therefore, their dip at zero may reflect an early developmental process. In contrast to leukocytes, the association of age with cytokine and chemokine levels was more heterogenic. Five different patterns of age-dependency could be identified (Figures 1D–H). The majority of parameters followed the pattern observed for immune cells, and showed a log linear decline with increasing age (Figures 1D,E). Some of them were associated with a dip at zero (ÍL-2, CCL11 (Eotaxin), IL-1β, FGF-b, G-CSF, IFN-γ, IL-13, IL-5, IL-4, IL-17, CCL5 (RANTES), and TNF-α, Figure 1D), while others (GM-CSF, CXCL1 (GRO- α), IL12-p70, IL-17, IL2-Rα (sCD25), IL-9, CXCL10 (IP-10), M-CSF, CCL4 (MIP-1 β) were not (Figure 1G). CXCL8 (IL-8), SCGF-b and HGF showed a steady decrease with age that was more extreme than log-linear (Figure 1E). IL-16 and VCAM1 were as well associated with a larger than log-linear decline in young age groups, but showed a steady log-linear increase from age three onwards (Figure 1H). About half of the examined soluble factors (CCL27 (CTACK), ICAM1, IFN-α2, IL-10, IL-12p40, IL-15, IL-18, IL-1α, IL-1RA, IL-3, IL-6, LIF, CCL2 (MCP1), CCL7 (MCP-3), MIF, CXCL9 (MIG), CCL3 (MIP-1α, PDGF-bb, SCF, CXCL12 (SDF-1α), TNF-β, TRAIL, and VEGF) showed no clear association with age (Figure 1F). Detailed information about the functional forms of all individual markers can be found in Supplementary Table 2.

### Effect of Primary Liver Disease on Pre-LTx Immune Markers

**Figure 2:**
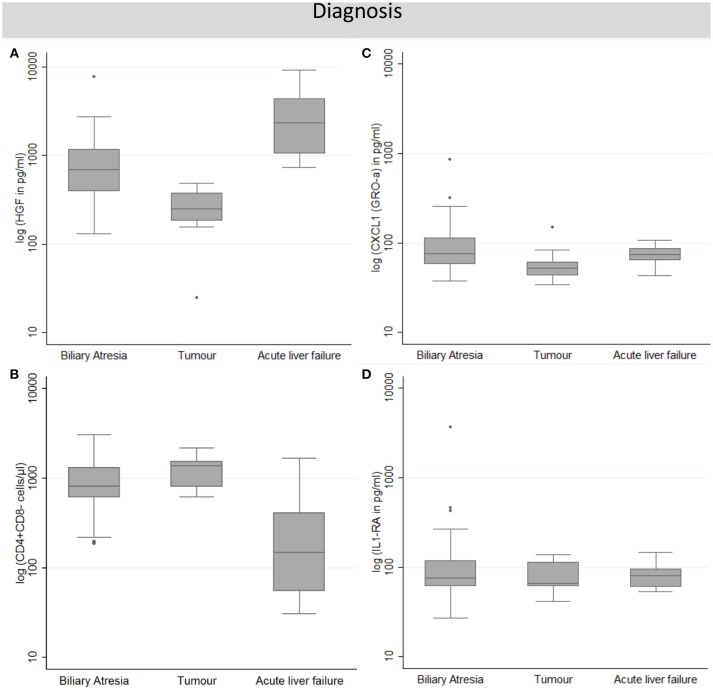
Overview of the functional patterns of the association of primary diagnosis and immune monitoring parameters identified in this study. Each pattern is visualized in this figure using one proxy example representative for this pattern. Patterns include those with higher values in acute liver failure and lower ones in the tumor group when compared to biliary atresia [**A**; granulocytes, HGF, LIF, M-SCF, VCAM, ICAM1, CXCL10 (IP-10), IL-2Rα (sCD25), and IL18], those with lower values in acute liver failure when compared to both other groups (**B**; Peripheral blood T cells, IL-4, IL-5, and IL-13), those with higher values in biliary atresia than in the other groups [**C**; CD19^+^ B cells, CCL11 (Eotaxin), IL-3, IL-6, CXCL8 (IL-8), IL-9, IL-16, MIF, CCL4 (MIP-1 β), and CXCL1 (GRO-α)] and those with no diagnosis-specific differences (**D**; monocytes and all soluble factors not described in **A–C**). Displayed are boxplots of log-transformed parameter values. Boxes represent the 25, 50, and 75% percentile; whiskers are defined as the upper/lower box boundary plus/minus 1.5 times the interquartile range.

There was a systematic effect of primary liver disease on immune markers in this study. In general, three different patterns could be differentiated (Figures 2A–C). Peripheral blood T cells were overall highest in tumor patients, and lowest in patients with acute liver failure indicating T cell expansion in tumor (Supplementary Table 3). In contrast, CD19^+^ B cells displayed lowest counts in tumor and highest counts in biliary atresia patients who also represent the youngest age group. This was not the case for granulocytes which showed highest levels in acute liver failure patients and lowest levels in tumor patients; monocytes counts were not different between groups. The patterns observed for immune cells could also be found in soluble immune markers. With respect to T cell-associated cytokines, TH2 cytokines (like IL-4, IL-5 and IL-13) were selectively elevated in tumor patients suggesting an immunosuppressed status in these patients. In contrast, levels of HGF, LIF, M-SCF, VCAM, and ICAM1 were highest for acute liver failure (ALF) and lowest for tumor patients, indicating that tissue damage plays the largest pathophysiological role in patients with ALF. The same was true for CXCL10 (IP-10), IL-2Rα (sCD25), and IL18 supporting the interpretation of liver damage as consequence of inflammation in ALF. CCL11 (Eotaxin) was selectively increased in biliary atresia patients, while IL-3, IL-6, CXCL8 (IL-8), IL-9, IL-16, MIF, CCL4 (MIP-1 β), and CXCL1 (GRO-α), were increased in both biliary atresia and acute liver failure. For all other plasma proteins, there was no relevant difference between diagnosis groups (Figure 2; Supplementary Table s3).

### Competing Effects of Age and Primary Liver Disease on Pre-LTx Immune Markers

Since age and underlying liver disease were associated with each other and with the outcome, their effects could be mutually confounded; thus, we aimed to disentangle the effect of both factors by using multivariable models. We found that age patterns were virtually unchanged when adjusting for underlying diagnoses. There were only minor changes in the functional form of the age effect on some markers (log linear decrease with dip at zero instead of without for lymphocytes, PDGF-bb; log linear decrease without dip at zero instead of with for CCL11 (Eotaxin); more than log linear decrease instead of log linear for monocytes). The same is true for the effect of underlying liver disease. There were no relevant changes of effect size when estimates were adjusted for age differences between groups (Supplementary Table 4). This suggests that the results of the univariable analyses for age and primary liver disease remain valid. When looking at the relative contribution of both factors to the explained variance in the respective multivariable regression methods, for all cellular and soluble immune markers more than 75% of the explained variance (measured by the r^2^ value) was attributable to differences in age; the explained variance attributable to underlying primary disease was low in relative and absolute (1–16%) numbers (data not shown). There was no evidence for center effects when adding transplant center as a random effect to the multivariable model (data not shown). Stability checks via MFPBOOT revealed an acceptable stability of all multivariable models.

## Discussion

Using data from the ChilSFree study, we show for the first time that primary disease and age affect immune markers in children with end-stage liver disease just before LTx. These differences in immune status in children with end-stage liver disease prior to the onset of immunosuppressive therapy and adaptive immune reactions to the graft might affect the patterns of immune reaction after transplantation. While it was expected from findings in healthy children, that age affects the immune system in children before liver transplantation, this study adds to the literature that the effects of age and primary diagnosis are independent and that both factors need to be taken into account when studying children with end-stage liver disease.

Our study represents the largest prospective cohort study of liver transplanted children which focuses on an assessment of the immune function. Patients enrolled in ChilSFree suffer from more than 10 different primary diagnostic entities, giving a representative picture of end-stage liver disease in European children. Comparison of immune status by primary diagnosis is, however, limited by the small numbers of patients in the majority of diagnostic subgroups. We, therefore, focused our analysis on the comparison of three distinct entities: (i) biliary atresia, which is the leading cause of LTx in ChilSFree and could serve as a model for biliary cirrhotic liver disease associated with chronic inflammation; (ii) acute liver failure which is characterized by an acute destruction of liver tissue; and (iii) liver tumors, mainly hepatoblastoma patients, who are typically treated with chemotherapy and immunomodulating drugs before LTx and suffer from tumor-mediated immunosuppression.

We found immune cell numbers to be highest in liver tumor patients, and lowest in patients with acute liver failure. This indicates a strong T cell expansion or maintenance after chemotherapy, especially of CD4^+^ T helper cells that are nevertheless unable to control tumor growth in these tumor patients. Since all but one liver tumor patients were patients with hepatoblastoma, this disease group was very homogenous. Immune cell numbers and cytokine levels in these patients obviously do not only reflect the effects of the underlying disease, but also the effects of pre-transplant chemotherapy that seems to reduce primarily CD8^+^ T cells and B cells. Cytokine analysis in tumor patients revealed comparatively lower levels in IL-16, IL-18, CXCL8 (IL-8), IL-9, and CXCL10 (IP-10), indicating that the higher T cell numbers were not accompanied by increased T cell activity. Our findings, therefore, cannot necessarily be used to delineate the pathophysiological role of the immune system in this entity. However, they are a reminder that the immune composition in these patients differs from other liver transplant candidates, which should be taken into account for post-transplant immunosuppression.

In patients with acute liver failure (ALF), levels for HGF, ICAM-1, and IFN-α2 were found to be elevated compared to patients with biliary atresia or liver tumor patients. This probably reflects a more intense inflammation and acute tissue damage in these patients which is supported by cytokines associated with cell damage like IL-18.

In children with biliary atresia, serum levels of CCL11 (Eotaxin), IL-16, and GRO-α (CXCL1), as well as numbers of B lymphocytes, were higher than in children with ALF or liver tumors. Immune-mediated destruction of the bile ducts is thought to be the main pathophysiological process in the development of biliary atresia, although the origin of the immune reaction (immune dysregulation triggered by virus infection vs. immune reaction against maternal antigens) still remains elusive (12). Published data on cytokine and chemokine levels in biliary atresia patients have largely concentrated on the immunological processes in the early stages of disease, namely at diagnosis or at the time of Kasai operation (13–16). A rather pro-inflammatory cytokine profile has been described (14, 16). In our study, pro-inflammatory cytokines were not increased in the BA subgroup compared to other disease etiologies. This is most likely an effect of time and disease stage, since our analysis was made in a situation of advanced end-stage liver disease just prior to LTx. One study that compared cytokine levels in biliary atresia children with advanced cirrhosis with those in healthy children found anti-inflammatory cytokines IL-10 and IL-6 to be elevated (17). Both cytokines correlated with disease stage and displayed a negative correlation with nutritional parameters. Since comparison in this study was made with healthy children, it remains unclear whether the observed differences are specific to biliary atresia, or are effects of advanced cirrhosis *per se*. In our study, IL6 and IL10 levels did not differ between disease groups. Moreover, we did not incorporate an analysis of nutritional status. The clinical relevance of the elevation of CXCL1 (GRO-α) in biliary atresia remains unclear. Reduced levels of CXCL1 have been associated with advanced stages of hepatic fibrosis in adults with chronic hepatitis C virus infection (18). To the best of our knowledge, no data on the role of this chemokine in advanced liver cirrhosis in children are available.

The effect of age on immune cell numbers has previously been demonstrated in healthy children (3). The patterns of age dependency for lymphocyte subsets described for healthy children correspond to our findings in children with liver disease, in that there is a log linear decrease with age. The dip at zero seen in CD8^+^CD56^+^ cells could possibly be explained by the fact that CD56 may also represent a thymic output marker for cytotoxic T cells. The delayed increase in the first months of life could, therefore, be associated with T cell development and maturation. Unfortunately, similar data do not exist regarding chemokine and cytokine plasma levels. Therefore, it is impossible to interpret our data based on studies in healthy children. Nevertheless, our analyses demonstrate that despite a strong association of age and underlying liver disease, both parameters affect immune markers independently.

Although ChilSFree represents one of the largest studies on pediatric liver transplantation in Europe, our study has several limitations, For instance, the sample size is still small in absolute numbers given the high heterogeneity of the study population. This precludes a meaningful analysis of immune status variations in smaller diagnostic subgroups. Moreover, the form of association between immune markers and age underlies a considerable level of uncertainty so that there might be some misclassification with respect to the groups described in Figure 1. Several statistical tests were conducted for the association of primary diagnosis and immune markers without correcting for multiple testing. However, given that this is an exploratory analysis aiming at the identification of consistent patterns and not a confirmatory study *p*-values should only be compared on a relative scale and not interpreted in absolute numbers. Pathway analyses (like Ingenuity) were not performed because we did not focus on specific pathways and we did not measure a complete set of cytokines and chemokines; such protein assays are not available (in contrast to mRNA profiling). Therefore, pathway analysis programs would rather give a scattered picture without providing reliable networks. Another potential limitation of our analysis results from the fact that the practice of listing for transplantation as well as availability of organs might differ between centers. Participating centers are organized in four different transplant organizations (UK transplant, Swiss Transplant, Agence de Biomédicine (France), Eurotransplant). Since disease severity at time of LTx might differ between these centers, this can potentially impact levels of immune markers. In order to avoid further center effects, sample collection and shipping from the participating centers were harmonized a priori and high quality control measures were taken. There might, however, still be the potential for center effects due to unforeseen sample handling issues. Nevertheless, center of origin had no clear additional effect on immune parameters in our study once they were adjusted for age and diagnosis.

In summary, we show for the first time that age and underlying diagnosis are major independent determinants of cellular and soluble immune marker levels in children with end stage liver disease. The results of this analysis need to be taken into account when designing future studies aiming to assess the role of immune monitoring parameters as a biomarker after liver transplantation.

## Data Availability Statement

The raw data supporting the conclusions of this manuscript will be made available by the authors, without undue reservation, to any qualified researcher.

## Author Contributions

UB, IG, CF, TM, AK, and RM developed the study design and initiated the study. UB and IG initiated the international cooperation and facilitated development and implementation of the eCRF. IG and TL are in charge of patient enrolment and data acquisition at Hannover Medical School. IG, TL, and UB provide analysis and interpretation of clinical data. LD, PM, DK, DD, VM, JP, PC, and LH are principal investigators at the international cooperating centers. They have made important contributions toward study design and study layout and are in charge of local data acquisition and documentation. TM, AK, and RM provide the statistical concepts for the study and made important contributions toward study design. CF provided analysis, documentation, and interpretation of immunological data. All authors have read and approved the final manuscript.

### Conflict of Interest Statement

The authors declare that the research was conducted in the absence of any commercial or financial relationships that could be construed as a potential conflict of interest.

## Abbreviations

LTx: Liver transplantation
ALF: Acute liver failure
BA: Biliary atresia
pLTx: Pediatric liver transplantation.
