# Supplementary material for: Immune Status in Children Before Liver Transplantation—A Cross-Sectional Analysis Within the ChilsSFree Multicentre Cohort Study
Source: Front Immunol. 2019 Jan 25;10:52. doi: 10.3389/fimmu.2019.00052 (PMC6357985; doi:10.3389/fimmu.2019.00052)
Supplement: Supplementary file 1 [file Table_1.DOCX]

**SUPPLEMENTARY MATERIAL**

Supplementary Table 1: Immune monitoring markers stratified by sex. Back-transformed means and confidence intervals of the natural logarithms of the original marker values are displayed.

|  | Sex | | | | | |  |
| --- | --- | --- | --- | --- | --- | --- | --- |
| **Marker** | **Male** | | | **Female** | | | **p value**** |
|  | Mean | 95% Confidence Interval | | Mean | 95% Confidence Interval | |  |
| CD3^+^ T cells | 1387 | 1165 | 1653 | 1298 | 1070 | 1574 | 0.610 |
| CD8^+^ cytotoxic T cells | 437 | 364 | 525 | 392 | 322 | 477 | 0.420 |
| CD4^+^T helper cells | 776 | 637 | 944 | 716 | 569 | 902 | 0.600 |
| CD19^+^ B cells | 466 | 349 | 621 | 379 | 266 | 540 | 0.366 |
| CD16+56^+^ NK cells | 158 | 128 | 195 | 145 | 117 | 179 | 0.574 |
| granulocytes | 2518 | 2136 | 2968 | 2203 | 1826 | 2657 | 0.285 |
| all lymphocytes | 2330 | 1956 | 2775 | 2175 | 1821 | 2598 | 0.585 |
| monocytes | 443 | 379 | 518 | 437 | 369 | 518 | 0.904 |
| CTACK CCL27 | 446 | 363 | 547 | 479 | 395 | 579 | 0.615 |
| Eotaxin CCL11 | 62 | 54 | 71 | 62 | 54 | 70 | 0.996 |
| FGFb | 25 | 22 | 27 | 23 | 20 | 27 | 0.448 |
| G-CSF | 96 | 85 | 110 | 89 | 78 | 102 | 0.409 |
| GM-CSF | 1 | 0 | 1 | 1 | 1 | 2 | 0.292 |
| GRO-α CXCL1 | 71 | 64 | 79 | 74 | 65 | 84 | 0.691 |
| HGF | 601 | 478 | 755 | 718 | 546 | 945 | 0.317 |
| ICAM1 | 47215 | 43338 | 51440 | 44649 | 39439 | 50547 | 0.452 |
| IFN-α2 | 61 | 59 | 64 | 61 | 57 | 66 | 0.967 |
| IFN-γ | 202 | 183 | 224 | 179 | 151 | 212 | 0.206 |
| IL-10 | 9 | 7 | 11 | 9 | 7 | 11 | 0.862 |
| IL-12p40 | 5724 |  |  | 5601 |  |  | 0.101 |
| IL-12p70 | 19 | 16 | 22 | 18 | 15 | 21 | 0.648 |
| IL-13 | 6 | 4 | 7 | 5 | 4 | 7 | 0.688 |
| IL-15 | 5894 |  |  | 5432 |  |  | 0.289 |
| IL-16 | 629 | 550 | 721 | 614 | 537 | 702 | 0.797 |
| IL-17 | 54 | 48 | 61 | 54 | 47 | 62 | 0.985 |
| IL-18 | 114 | 89 | 145 | 139 | 109 | 177 | 0.252 |
| IL-1α | 5960 |  |  | 5365 |  |  | 0.129 |
| IL-1β | 4 | 3 | 4 | 3 | 2 | 5 | 0.728 |
| IL-1RA | 88 | 75 | 103 | 86 | 74 | 100 | 0.889 |
| IL-2 | 4 | 3 | 6 | 5 | 3 | 7 | 0.706 |
| IL-2Rα sCD25 | 146 | 130 | 165 | 147 | 123 | 176 | 0.972 |
| IL-3 | 322 | 298 | 347 | 320 | 281 | 365 | 0.953 |
| IL-4 | 3 | 2 | 3 | 2 | 2 | 3 | 0.204 |
| **IL-5** | **20** | **17** | **22** | **15** | **11** | **19** | **0.035** |
| **IL-6** | **10** | **8** | **12** | **14** | **11** | **19** | **0.030** |
| IL-7 | 5 | 4 | 7 | 4 | 3 | 6 | 0.271 |
| IL-8 | 38 | 32 | 46 | 43 | 33 | 57 | 0.443 |
| IL-9 | 47 | 42 | 53 | 49 | 43 | 55 | 0.695 |
| IP-10 CXCL10 | 768 | 625 | 944 | 812 | 641 | 1029 | 0.722 |
| LIF | 28 | 25 | 30 | 27 | 23 | 31 | 0.817 |
| MCP-1 CCL2 | 7 | 6 | 9 | 7 | 5 | 9 | 0.866 |
| MCP-3 CCL7 | 56 | 50 | 63 | 60 | 51 | 70 | 0.573 |
| M-CSF | 8 | 6 | 10 | 8 | 6 | 10 | 0.956 |
| MIF | 4101 | 3258 | 5164 | 3599 | 2855 | 4538 | 0.429 |
| MIG CXCL9 | 617 | 528 | 721 | 615 | 505 | 750 | 0.983 |
| MIP1a CCL3 | 1 | 1 | 2 | 1 | 1 | 2 | 0.955 |
| MIP1b CCL4 | 56 | 49 | 64 | 63 | 55 | 73 | 0.223 |
| PDGF-BB | 295 | 230 | 377 | 218 | 166 | 285 | 0.100 |
| RANTES CCL5 | 3127 | 2823 | 3464 | 2780 | 2454 | 3148 | 0.144 |
| SCF | 73 | 63 | 84 | 71 | 61 | 84 | 0.848 |
| SCGFb | 24280 | 19869 | 29670 | 25796 | 19633 | 33895 | 0.718 |
| SDF-1α CXCL12 | 365 | 348 | 384 | 342 | 321 | 365 | 0.108 |
| TNF-α | 23 | 20 | 27 | 22 | 18 | 26 | 0.587 |
| TNF−β | 0 | 0 | 1 | 0 | 0 | 1 | 0.327 |
| TRAIL | 44 | 37 | 53 | 53 | 45 | 63 | 0.155 |
| VCAM1 | 31452 | 28700 | 34468 | 31923 | 28199 | 36138 | 0.846 |
| VEGF | 21 | 17 | 27 | 19 | 15 | 23 | 0,402 |

*displayed are medians only due to unusual parameter distributions on the log scale. P values are obtained via Wilcoxon ranksum tests.

**p values are obtained via two-sided t tests on the log scale.

Supplementary Table 2: Association between immune monitoring markers and patients' age. For each parameter the equation derived from the fractional polynomial linear regression model as well as the corresponding p values are displayed. The equation can be used for estimating parameter values based on patients' age. P values are obtained via t tests and compare a model with age against an empty model.

| Cellular markers | | | |
| --- | --- | --- | --- |
|  | **Variable** | **Equation** | **p value** |
| Loglinear decline with increasing age | CD3^+^ T cells | 3.128665-0.0378527*(Age-5.429570703) | <0.001 |
|  | CD4^-^CD8^-^ T cells | 2.000567-0.0283192*(Age-5.429570703) | <0.001 |
|  | CD8^+^ cytotoxic T cells | 2.618423-0.036272*(Age-5.429570703) | <0.001 |
|  | CD4^+^CD56^+^ T cells | 0.8565651-0.2113175*(ln(Age/10)+0.6107250224) | <0.001 |
|  | CD4^+^T helper cells | 2.873496-0.0403158*(Age-5.429570703) | <0.001 |
|  | all lymphocytes | 3.353414-0.0397219*(Age-5.429570703) | <0.001 |
|  | monocytes | 2.643728-0.0147837*(Age-5.429570703) | 0.001 |
|  | CD19^+^ B cells | 2.626485-0.0569551*(Age-5.429570703) | <0.001 |
|  | CD16+56^+^ NK cells | 2.180752-0.0323915*(Age-5.429570703) | <0.001 |
| Loglinear decline with increasing age with additional dip at zero | CD8^+^CD56^+^ | 1.148066-0.0534598*(Age/10^-1-1.841766237)+0.3089752*(Age/10^-0.5-1.357116884) | <0.001 |
| No association | granulocytes | 3.373944+0.002389*(Age-5.429570703) | 0.632 |

| Soluble markers | | | |
| --- | --- | --- | --- |
|  | **Variable** | **Equation** | **p value** |
| Loglinear decline with increasing age with additional dip at zero | Eotaxin CCL11 | 1.707207-0.0004073*(Age/10^-2-3.39210287)+0.0261913*(Age/10^-1-1.841766237) | <0.001 |
|  | FGFb | 1.464966+0.2698113*(ln(Age/10)+0.6107250224)-0.8891145*(Age/10^0.5-0.7368562073) | <0.001 |
|  | G-CSF | 2.022956-0.2294649*(Age/10^-0.5-1.357116884)-0.2282536*(ln(Age/10)+0.6107250224) | <0.001 |
|  | IFN-γ | 2.380315+0.2944547*(ln(Age/10)+0.6107250224)-0.8885748*(Age/10^0.5-0.7368562073) | <0.001 |
|  | IL-7 | 0.8803753+1.203388*(Age/10^0.5-0.7368562073)-0.833878*(Age/10^0.5*ln(Age/10)+0.4500165237) | 0.001 |
|  | IL-13 | 0.9372427+1.272431*(Age/10^0.5-0.7368562073)-0.9221089*(Age/10^0.5*ln(Age/10)+0.4500165237) | <0.001 |
|  | IL-1β | 0.6096287-0.0134045*(Age/10^-1-1.841766237) | 0.007 |
|  | IL-4 | 0.486653+0.2352579*(ln(Age/10)+0.6107250224)-0.7459225*(Age/10^0.5-0.7368562073) | <0.001 |
|  | IL-5 | 1.411479+1.136069*(Age/10^0.5-0.7368562073)-0.7901217*(Age/10^0.5*ln(Age/10)+0.4500165237) | <0.001 |
|  | IL-2 | 0.7068029-0.0007343*(Age/10^-2-3.39210287) | <0.001 |
|  | RANTES CCL5 | 3.514941-0.0138238*(Age/10^-1-1.841766237)-0.2394685*(Age/10^0.5-0.7368562073) | <0.001 |
|  | TNF-α | 1.462768+0.3472087*(ln(Age/10)+0.6107250224)-1.109805*(Age/10^0.5-0.7368562073) | <0.001 |
| Loglinear decline with increasing age without additional dip at zero | GM-CSF | -0.0947638-0.0651845*(Age-5.429570703) | 0.003 |
|  | GROα CXCL1 | 1.859982-0.0099205*(Age-5.429570703) | 0.002 |
|  | IL-12p70 | 1.268768-0.0089888*(Age-5.429570703) | 0.048 |
|  | IL-17 | 1.732084-0.0113053*(Age-5.429570703) | 0.002 |
|  | IL-2Rα CD25 | 2.166015-0.0095926*(Age-5.429570703) | 0.023 |
|  | IL-9 | 1.677616-0.0137651*(Age-5.429570703) | <0.001 |
|  | IP-10 CXCL10 | 2.896721-0.0173533*(Age-5.429570703) | 0.005 |
|  | M-CSF | 0.9079153-0.0139443*(Age-5.429570703) | 0.036 |
|  | MIP-1β CCL4 | 1.773817-0.0134393*(Age-5.429570703) | 0.001 |
| Decline with increasing age with following increase in higher age | VCAM1 | 4.423232-0.4816675*(Age/10^0.5-0.7368562073)+0.3702918*(Age/10^0.5*ln(Age/10)+0.4500165237) | <0.001 |
|  | IL-16 | 2.702406-0.9523606*(Age/10^0.5-0.7368562073)+0.6108106*(Age/10-0.5429570703) | 0.003 |
| More extreme than loglinear decline with increasing age with additional dip at zero | IL-8 CXCL8 | 1.491225+0.1350111*(Age/10^-0.5-1.357116884) | <0.001 |
|  | SCGFb | 4.264725+0.154336*(Age/10^-0.5-1.357116884) | <0.001 |
|  | HGF | 2.735799+0.0164119*(Age/10^-1-1.841766237) | 0.001 |
| No association | ICAM1 | 4.662755+0.0009358*(Age-5.429570703) | 0.752 |
|  | IFNα2 | 1.78783-0.0018908*(Age-5.429570703) | 0.249 |
|  | IL-10 | 0.9421049-0.0043657*(Age-5.429570703) | 0.427 |
|  | IL-12p40 | 1.407409+0.0046166*(Age-5.429570703) | 0.617 |
|  | IL-15 | -0.8894382+0.0086536*(Age-5.429570703) | 0.176 |
|  | IL-18 | 2.096312-0.0046784*(Age-5.429570703) | 0.502 |
|  | IL-1a | -0.4705767+0.0003511*(Age-5.429570703) | 0.818 |
|  | IL-1RA | 1.938898-0.003051*(Age-5.429570703) | 0.493 |
|  | IL-3 | 2.506414-0.0019849*(Age-5.429570703) | 0.497 |
|  | IL-6 | 1.071742-0.0114346*(Age-5.429570703) | 0.105 |
|  | LIF | 1.436223-0.0044742*(Age-5.429570703) | 0.170 |
|  | MCP1 CCL2 | 0.8528317+0.0030537*(Age-5.429570703) | 0.619 |
|  | MCP3 CCL7 | 1.76237-0.0025617*(Age-5.429570703) | 0.504 |
|  | MIF | 3.586464-0.0002189*(Age-5.429570703) | 0.973 |
|  | MIG CXCL9 | 2.789755-0.006136*(Age-5.429570703) | 0.217 |
|  | MIP-1α CCL3 | 0.044457-0.0069705*(Age-5.429570703) | 0.492 |
|  | PDGFbb | 2.407789-0.0103172*(Age-5.429570703) | 0.161 |
|  | SCF | 1.858411-0.0046539*(Age-5.429570703) | 0.269 |
|  | SDF1α CXCL12 | 2.549501+0.000776*(Age-5.429570703) | 0.631 |
|  | TNF-β | -0.4077239-0.0239956*(Age-5.429570703) | 0.183 |
|  | TRAIL | 1.683178+0.0032006*(Age-5.429570703) | 0.522 |
|  | VEGF | 1.303894-0.0120264*(Age-5.429570703) | 0.050 |
|  | CTACK | 2.663378-0.008087*(Age-5.429570703) | 0.151 |

Supplementary Table 3: Association between immune monitoring markers and underlying diagnosis of liver disease. Back-transformed means and confidence intervals of the natural logarithms of the original marker values as well as the corresponding p value are displayed. P values are obtained via oneway ANOVAs.

| Parameter | **Biliary atresia** | | | **Tumour** | | | **Acute Liver Failure** | | | **Other Diseases** | | | **p value** |
| --- | --- | --- | --- | --- | --- | --- | --- | --- | --- | --- | --- | --- | --- |
|  | Mean | 95% Confidence Interval | | Mean | 95% Confidence Interval | | Mean | 95% Confidence Interval | | Mean | 95% Confidence Interval | |  |
| **CD3^+^ T cells** | **1394** | **1115** | **1742** | **2067** | **1324** | **3229** | **647** | **375** | **1117** | **1306** | **1102** | **1547** | **0.015** |
| CD8^+^ cytotoxic T cells | 437 | 345 | 552 | 669 | 419 | 1071 | 293 | 165 | 521 | 382 | 320 | 457 | 0.095 |
| **CD4^+^T helper cells** | **797** | **625** | **1017** | **1184** | **727** | **1928** | **166** | **91** | **301** | **766** | **637** | **922** | **<0.001** |
| **CD19^+^ B cells** | **591** | **407** | **857** | **94** | **45** | **198** | **265** | **106** | **659** | **454** | **342** | **603** | **<0.001** |
| CD16+56^+^ NK cells | 152 | 117 | 196 | 149 | 89 | 250 | 67 | 35 | 126 | 164 | 134 | 199 | 0.072 |
| **granulocytes** | **2081** | **1692** | **2559** | **1170** | **773** | **1770** | **3183** | **1917** | **5285** | **2763** | **2360** | **3234** | **0.001** |
| all lymphocytes | 2417 | 1948 | 2999 | 2494 | 1620 | 3839 | 1156 | 681 | 1961 | 2257 | 1916 | 2660 | 0.082 |
| monocytes | 409 | 334 | 500 | 481 | 321 | 719 | 483 | 295 | 791 | 450 | 386 | 524 | 0.820 |
| CTACK CCL27 | 528 | 413 | 674 | 439 | 269 | 716 | 678 | 373 | 1235 | 416 | 345 | 501 | 0.258 |
| **Eotaxin CCL11** | **82** | **70** | **95** | **40** | **29** | **54** | **53** | **36** | **78** | **57** | **51** | **64** | **<0.001** |
| FGFb | 23 | 20 | 27 | 26 | 19 | 35 | 24 | 17 | 35 | 25 | 22 | 27 | 0.866 |
| G-CSF | 85 | 72 | 100 | 101 | 73 | 140 | 76 | 51 | 114 | 99 | 87 | 112 | 0.334 |
| GM-CSF | 1 | 0 | 3 | 1 | 0 | 5 | 1 | 0 | 15 | 1 | 0 | 1 | 0.916 |
| **GRO-α CXCL1** | **84** | **73** | **96** | **55** | **42** | **73** | **73** | **52** | **102** | **69** | **62** | **77** | **0.036** |
| **HGF** | **690** | **517** | **920** | **225** | **126** | **399** | **2295** | **1134** | **4648** | **661** | **531** | **823** | **<0.001** |
| **ICAM1** | **53151** | **47283** | **59748** | **29965** | **23713** | **37864** | **77445** | **58148** | **103145** | **42857** | **39209** | **46845** | **<0.001** |
| **IFN-α2** | **63** | **59** | **68** | **55** | **48** | **63** | **78** | **66** | **93** | **60** | **57** | **63** | **0.010** |
| IFN-γ | 171 | 145 | 203 | 210 | 150 | 295 | 182 | 120 | 275 | 200 | 176 | 227 | 0.489 |
| IL-10 | 8 | 6 | 10 | 8 | 5 | 13 | 12 | 7 | 21 | 9 | 7 | 11 | 0.690 |
| IL-12p40 | 29 | 20 | 44 | 13 | 6 | 28 | 40 | 15 | 106 | 25 | 18 | 34 | 0.240 |
| IL-12p70 | 19 | 16 | 24 | 19 | 12 | 28 | 14 | 9 | 23 | 19 | 16 | 22 | 0.739 |
| **IL-13** | **5** | **3** | **6** | **9** | **5** | **18** | **2** | **1** | **5** | **6** | **5** | **8** | **0.041** |
| IL-15 | 0 | 0 | 0 | 0 | 0 | 0 | 0 | 0 | 0 | 0 | 0 | 0 | 0.173 |
| **IL-16** | **769** | **654** | **905** | **437** | **316** | **605** | **740** | **497** | **1101** | **576** | **509** | **652** | **0.005** |
| IL-17 | 54 | 46 | 64 | 41 | 30 | 56 | 61 | 41 | 90 | 55 | 49 | 63 | 0.323 |
| **IL-18** | **160** | **119** | **215** | **101** | **56** | **182** | **406** | **196** | **838** | **103** | **82** | **129** | **0.001** |
| IL-1α | 0 | 0 | 0 | 0 | 0 | 0 | 0 | 0 | 0 | 0 | 0 | 0 | 0.411 |
| IL-1β | 4 | 3 | 5 | 4 | 2 | 8 | 3 | 1 | 7 | 4 | 3 | 5 | 0.962 |
| IL-1RA | 91 | 75 | 111 | 77 | 52 | 113 | 80 | 50 | 128 | 87 | 75 | 101 | 0.858 |
| IL 2 | 4 | 2 | 7 | 5 | 2 | 13 | 3 | 1 | 11 | 5 | 3 | 7 | 0.953 |
| IL-2Rα sCD25 | 145 | 120 | 174 | 113 | 78 | 163 | 240 | 153 | 376 | 148 | 128 | 170 | 0.089 |
| **IL-3** | **350** | **309** | **397** | **269** | **210** | **345** | **443** | **327** | **600** | **303** | **276** | **333** | **0.024** |
| IL-4 | 2 | 2 | 3 | 3 | 2 | 4 | 3 | 2 | 3 | 3 | 2 | 3 | 0.241 |
| IL-5 | 13 | 11 | 17 | 25 | 15 | 41 | 14 | 8 | 26 | 19 | 15 | 22 | 0.074 |
| IL-6 | 13 | 10 | 18 | 6 | 3 | 10 | 11 | 5 | 23 | 13 | 10 | 16 | 0.087 |
| IL-7 | 4 | 3 | 5 | 8 | 4 | 14 | 4 | 2 | 8 | 5 | 4 | 7 | 0.161 |
| **IL-8 CXCL8** | **61** | **47** | **80** | **18** | **11** | **31** | **47** | **25** | **90** | **36** | **29** | **44** | **<0.001** |
| **IL-9** | **55** | **48** | **65** | **33** | **25** | **45** | **52** | **36** | **75** | **46** | **41** | **51** | **0.020** |
| *IP-10 CXCL10* | *1007* | *787* | *1287* | *285* | *174* | *466* | *2480* | *1359* | *4526* | *709* | *588* | *855* | *<0.001* |
| **LIF** | **30** | **26** | **34** | **22** | **16** | **28** | **43** | **31** | **61** | **26** | **23** | **29** | **0.006** |
| MCP-1 CCL2 | 6 | 5 | 8 | 7 | 4 | 11 | 8 | 4 | 16 | 8 | 6 | 9 | 0.591 |
| MCP-3 CCL7 | 64 | 54 | 75 | 51 | 37 | 72 | 64 | 42 | 96 | 55 | 48 | 62 | 0.455 |
| **M-CSF** | **8** | **6** | **11** | **4** | **2** | **7** | **20** | **10** | **40** | **8** | **7** | **10** | **0.006** |
| MIF | 5196 | 3912 | 6901 | 2696 | 1528 | 4755 | 3913 | 1953 | 7843 | 3473 | 2799 | 4310 | 0.084 |
| **MIG CXCL9** | **588** | **480** | **721** | **470** | **312** | **706** | **1885** | **1145** | **3105** | **591** | **506** | **690** | **<0.001** |
| MIP1a CCL3 | 1 | 1 | 2 | 1 | 0 | 2 | 1 | 0 | 3 | 1 | 1 | 2 | 0.734 |
| **MIP1b CCL4** | **72** | **61** | **84** | **29** | **21** | **40** | **81** | **55** | **121** | **58** | **51** | **66** | **<0.001** |
| PDGF-BB | 294 | 213 | 407 | 252 | 132 | 482 | 252 | 114 | 555 | 240 | 187 | 307 | 0.800 |
| RANTES CCL5 | 2999 | 2603 | 3455 | 3516 | 2649 | 4667 | 2887 | 2041 | 4084 | 2899 | 2603 | 3229 | 0.655 |
| **SCF** | **81** | **68** | **97** | **46** | **32** | **66** | **109** | **70** | **168** | **69** | **61** | **80** | **0.011** |
| **SCGFb** | **36836** | **28121** | **48253** | **10361** | **6038** | **17779** | **59295** | **30607** | **114873** | **21178** | **17247** | **26004** | **<0.001** |
| SDF-1α CXCL12 | 349 | 326 | 375 | 350 | 304 | 403 | 385 | 323 | 457 | 354 | 336 | 374 | 0.790 |
| TNF-α | 21 | 17 | 25 | 26 | 18 | 40 | 19 | 12 | 31 | 23 | 20 | 27 | 0.581 |
| TNF−β | 0 | 0 | 1 | 1 | 0 | 3 | 4 | 1 | 27 | 0 | 0 | 1 | 0.079 |
| TRAIL | 44 | 36 | 55 | 55 | 35 | 85 | 56 | 33 | 96 | 49 | 41 | 58 | 0.743 |
| **VCAM1** | **38264** | **33933** | **43147** | **19214** | **15110** | **24431** | **47868** | **35667** | **64242** | **29572** | **26991** | **32401** | **<0.001** |
| VEGF | 21 | 16 | 28 | 22 | 13 | 37 | 12 | 6 | 24 | 21 | 17 | 25 | 0.483 |

Supplementary Table 4: Results of the multivariable regression models assessing the association between immune monitoring markers on the one hand and the combination of underlying diagnosis of liver disease, age and sex on the other hand. For each parameter the equation derived from the fractional polynomial linear regression model as well as the corresponding p values are displayed. This equation can be used for estimating parameter values based on the three independent variables.

| Parameter | Formula (DG2 stands for tumour (yes/no), DG3 for acute liver failure (yes/no), DG4 for other diseases (yes/no)) | p-value | | | | | |
| --- | --- | --- | --- | --- | --- | --- | --- |
|  |  | Second polynomial for Age in equation | First polynomial for Age in equation | Tumor vs. Biliary atresia | Acute liver failure vs. Biliary atresis | Other diseases vs. Biliary atresia | Sex |
| CD3^+^ T cells | 3.07959-0.0413113*(Age-5.429570703)-0.0632337*(sex-1)+0.2043158*DG2-0.2149249*DG3+0.1348194*DG4 |  | <0.001 | 0.017 | 0.035 | 0.008 | 0.147 |
| CD4^-^CD8^-^ T cells | 1.945818-0.0312336*(Age-5.429570703)-0.0429405*(sex-1)+0.1968078*DG2-0.110054*DG3+0.1187424*DG4 |  | <0.001 | 0.098 | 0.436 | 0.092 | 0.480 |
| CD8^+^ cytotoxic T cells | 2.590216-0.038958*(Age-5.429570703)-0.0832623*(sex-1)+0.2137111*DG2-0.0572128*DG3+0.0969815*DG4 |  | <0.001 | 0.026 | 0.613 | 0.086 | 0.089 |
| CD4^+^CD56^+^ T cells | 0.8644059-0.2079181*(ln(Age/10)+0.6107250224)+0.0026252*(sex-1)+0.1546667*DG2-0.1643866*DG3-0.0192841*DG4 |  | <0.001 | 0.316 | 0.370 | 0.832 | 0.973 |
| CD4^+^T helper cells | 2.745951-0.6835796*(Age/10^0.5-0.7368562073)-0.0528819*(sex-1)+0.2517341*DG2-0.5252904*DG3+0.1726721*DG4 |  | <0.001 | 0.007 | <0.001 | 0.002 | 0.264 |
| CD19^+^ B cells | 2.713045-0.0649833*(Age-5.429570703)-0.1943257*(sex-1)-0.7580019*DG2-0.1462895*DG3+0.1327441*DG4 |  | <0.001 | <0.001 | 0.409 | 0.132 | 0.012 |
| CD16+56^+^ NK cells | 2.12923-0.0377755*(Age-5.429570703)-0.0748021*(sex-1)+0.021527*DG2-0.2449872*DG3+0.1788544*DG4 |  | <0.001 | 0.847 | 0.067 | 0.007 | 0.192 |
| CD8^+^CD56^+^ | 1.143918-0.0540847*(Age/10^-1-1.841766237)+0.3165829*(Age/10^-0.5-1.357116884)-0.0425896*(sex-1)+0.0867428*DG2+0.1526068*DG3+0.0099035*DG4 | 0.001 | <0.001 | 0.549 | 0.374 | 0.908 | 0.560 |
| granulocytes | 3.348958-0.0030701*(Age-5.429570703)-0.0822323*(sex-1)-0.2573458*DG2+0.2063237*DG3+0.133437*DG4 |  | <0.001 | 0.013 | 0.092 | 0.028 | 0.118 |
| all lymphocytes | 3.252725-0.0001606*(Age/10^-2-3.39210287)-0.7203552*(Age/10^0.5-0.7368562073)-0.0766337*(sex-1)+0.0764747*DG2-0.1659969*DG3+0.1587738*DG4 | <0.001 | <0.001 | 0.311 | 0.065 | 0.001 | 0.048 |
| monocytes | 2.480622+0.0919691*(Age/10^-0.5-1.357116884)-0.0153735*(sex-1)+0.1509227*DG2+0.1645473*DG3+0.1285846*DG4 |  | <0.001 | 0.111 | 0.142 | 0.021 | 0.745 |
| CTACK CCL27 | 2.700704-0.0058937*(Age-5.429570703)+0.0186909*(sex-1)-0.0720615*DG2+0.1211294*DG3-0.0855063*DG4 |  | <0.001 | 0.553 | 0.403 | 0.235 | 0.764 |
| Eotaxin CCL11 | 1.829241-0.0737341*(ln(Age/10)+0.6107250224)-0.0169669*(sex-1)-0.267647*DG2-0.1232479*DG3-0.0890142*DG4 |  | <0.001 | <0.001 | 0.162 | 0.043 | 0.650 |
| FGFb | 1.476872+0.2827062*(ln(Age/10)+0.6107250224)-0.9475741*(Age/10^0.5-0.7368562073)-0.0470207*(sex-1)-0.020829*DG2-0.0083691*DG3+0.0267263*DG4 | <0.001 | <0.001 | 0.774 | 0.921 | 0.526 | 0.195 |
| G-CSF | 2.008814-0.2437844*(Age/10^-0.5-1.357116884)-0.2521684*(ln(Age/10)+0.6107250224)-0.0505702*(sex-1)+0.0149246*DG2-0.0608008*DG3+0.0679159*DG4 | <0.001 | <0.001 | 0.848 | 0.506 | 0.137 | 0.197 |
| GM-CSF | -0.2267586-0.0659474*(Age-5.429570703)+0.1647322*(sex-1)-0.0285525*DG2+0.2978758*DG3+0.0759462*DG4 |  | 0.342 | 0.951 | 0.592 | 0.783 | 0.492 |
| GRO-α CXCL1 | 1.901867-0.0092363*(Age-5.429570703)-0.0018539*(sex-1)-0.1712245*DG2-0.0364111*DG3-0.046427*DG4 |  | <0.001 | 0.011 | 0.647 | 0.241 | 0.957 |
| HGF | 2.664091+0.1072008*(Age/10^-0.5-1.357116884)+0.0293025*(sex-1)-0.3871283*DG2+0.6220197*DG3+0.0782755*DG4 |  | <0.001 | 0.006 | <0.001 | 0.333 | 0.672 |
| ICAM1 | 4.747462+0.0024107*(Age-5.429570703)-0.0359728*(sex-1)-0.255797*DG2+0.1631699*DG3-0.1027298*DG4 |  | <0.001 | <0.001 | 0.019 | 0.003 | 0.226 |
| IFNα2 | 1.80071-0.0017551*(Age-5.429570703)-0.0082359*(sex-1)-0.0583796*DG2+0.097373*DG3-0.0174873*DG4 |  | <0.001 | 0.092 | 0.019 | 0.392 | 0.641 |
| IFNγ | 2.389244+0.3079957*(ln(Age/10)+0.6107250224)-0.9588878*(Age/10^0.5-0.7368562073)-0.0675983*(sex-1)+0.0000288*DG2-0.018954*DG3+0.0463737*DG4 | <0.001 | <0.001 | 1.000 | 0.841 | 0.324 | 0.095 |
| IL-10 | 0.9043719-0.0060758*(Age-5.429570703)-0.0038387*(sex-1)-0.0067518*DG2+0.1733216*DG3+0.0563794*DG4 |  | <0.001 | 0.955 | 0.224 | 0.425 | 0.950 |
| IL-12p40 | 1.422874+0.0073492*(Age-5.429570703)+0.1331078*(sex-1)-0.3523821*DG2+0.0910538*DG3-0.0992329*DG4 |  | <0.001 | 0.076 | 0.699 | 0.397 | 0.191 |
| IL-12p70 | 1.281003-0.0097192*(Age-5.429570703)-0.0314452*(sex-1)-0.0136255*DG2-0.0992239*DG3+0.016137*DG4 |  | <0.001 | 0.889 | 0.396 | 0.781 | 0.531 |
| IL-13 | 0.9358676+1.276494*(Age/10^0.5-0.7368562073)-0.9372675*(Age/10^0.5*ln(Age/10)+0.4500165237)-0.0354359*(sex-1)+0.1498119*DG2-0.4100887*DG3+0.0538959*DG4 | 0.001 | <0.001 | 0.354 | 0.031 | 0.565 | 0.660 |
| IL-15 | -0.9657555+0.0061984*(Age-5.429570703)+0.0628712*(sex-1)-0.0695914*DG2-0.0979809*DG3+0.1056774*DG4 |  | <0.001 | 0.612 | 0.549 | 0.194 | 0.371 |
| IL-16 | 2.896184+0.0011463*(Age-5.429570703)-0.0168814*(sex-1)-0.2483218*DG2-0.0169668*DG3-0.1346131*DG4 |  | <0.001 | 0.002 | 0.859 | 0.005 | 0.681 |
| IL-17 | 1.714622-0.0136312*(Age-5.429570703)-0.0240168*(sex-1)-0.1132497*DG2+0.0896922*DG3+0.0602686*DG4 |  | <0.001 | 0.142 | 0.328 | 0.186 | 0.542 |
| IL-18 | 2.173217+0.0008525*(Age-5.429570703)+0.0704583*(sex-1)-0.1928759*DG2+0.3905977*DG3-0.2107089*DG4 |  | <0.001 | 0.179 | 0.023 | 0.014 | 0.337 |
| IL-1α | -0.4909049+0.0001817*(Age-5.429570703)+0.0214485*(sex-1)+0.0025006*DG2+0.0587845*DG3+0.0127751*DG4 |  | <0.001 | 0.939 | 0.134 | 0.511 | 0.203 |
| IL-1β | 0.6820939-0.0146627*(Age/10^-1-1.841766237)-0.0242123*(sex-1)-0.0162579*DG2-0.1179849*DG3-0.0868913*DG4 |  | <0.001 | 0.919 | 0.530 | 0.342 | 0.762 |
| IL-1RA | 1.957539-0.0031523*(Age-5.429570703)-0.0117759*(sex-1)-0.0736017*DG2-0.047379*DG3-0.0086533*DG4 |  | <0.001 | 0.448 | 0.682 | 0.880 | 0.813 |
| IL 2 | 0.8662575-0.0348298*(Age/10^-1-1.841766237)+0.0513168*(sex-1)-0.1253403*DG2-0.2842204*DG3-0.1228776*DG4 |  | <0.001 | 0.600 | 0.313 | 0.370 | 0.668 |
| IL-2Rα sCD25 | 2.14563-0.0116807*(Age-5.429570703)-0.0256575*(sex-1)-0.099341*DG2+0.2540119*DG3+0.0489486*DG4 |  | <0.001 | 0.266 | 0.018 | 0.354 | 0.574 |
| IL-3 | 2.547214-0.0009306*(Age-5.429570703)-0.0103423*(sex-1)-0.1153968*DG2+0.105681*DG3-0.0592286*DG4 |  | <0.001 | 0.064 | 0.153 | 0.108 | 0.745 |
| IL-4 | 0.4891473+0.1345568*(ln(Age/10)+0.6107250224)-0.3045233*(Age/10-0.5429570703)-0.051314*(sex-1)+0.0207384*DG2+0.022007*DG3+0.0497933*DG4 | <0.001 | <0.001 | 0.695 | 0.722 | 0.106 | 0.054 |
| IL-5 | 1.410334+1.097631*(Age/10^0.5-0.7368562073)-0.8037827*(Age/10^0.5*ln(Age/10)+0.4500165237)-0.1407001*(sex-1)+0.1270579*DG2-0.0519694*DG3+0.1040226*DG4 | <0.001 | <0.001 | 0.266 | 0.696 | 0.116 | 0.014 |
| IL-6 | 1.036974-0.0112976*(Age-5.429570703)+0.1414307*(sex-1)-0.3498458*DG2-0.0949607*DG3+0.0033279*DG4 |  | <0.001 | 0.019 | 0.590 | 0.970 | 0.063 |
| IL-7 | 0.8569666+1.151761*(Age/10^0.5-0.7368562073)-0.8315923*(Age/10^0.5*ln(Age/10)+0.4500165237)-0.0978291*(sex-1)+0.1488131*DG2-0.0937355*DG3+0.1042718*DG4 | 0.001 | <0.001 | 0.341 | 0.607 | 0.249 | 0.210 |
| IL-8 | 1.618132-0.1315828*(ln(Age/10)+0.6107250224)+0.0173854*(sex-1)-0.4405931*DG2-0.0063342*DG3-0.1201972*DG4 |  | <0.001 | <0.001 | 0.965 | 0.100 | 0.779 |
| IL-9 | 1.717272-0.01361*(Age-5.429570703)-0.0090583*(sex-1)-0.2080717*DG2+0.0098767*DG3-0.0353229*DG4 |  | <0.001 | 0.004 | 0.908 | 0.404 | 0.805 |
| IP-10 CXCL10 | 2.978066-0.0174226*(Age-5.429570703)-0.0320767*(sex-1)-0.5345323*DG2+0.4425937*DG3-0.0863739*DG4 |  | <0.001 | <0.001 | 0.002 | 0.220 | 0.599 |
| LIF | 1.47699-0.003899*(Age-5.429570703)-0.0237031*(sex-1)-0.1396651*DG2+0.1765828*DG3-0.0511282*DG4 |  | <0.001 | 0.042 | 0.031 | 0.206 | 0.497 |
| MCP-1 CCL2 | 0.8002869+0.0003931*(Age-5.429570703)-0.0157075*(sex-1)+0.0300781*DG2+0.1279874*DG3+0.0926392*DG4 |  | <0.001 | 0.822 | 0.421 | 0.242 | 0.818 |
| MCP-3 CCL7 | 1.792049-0.0010965*(Age-5.429570703)+0.0195862*(sex-1)-0.0896234*DG2+0.0008281*DG3-0.0579772*DG4 |  | <0.001 | 0.281 | 0.993 | 0.239 | 0.645 |
| M-CSF | 0.9064118-0.0170607*(Age-5.429570703)-0.0448735*(sex-1)-0.3253363*DG2+0.4229494*DG3+0.0474037*DG4 |  | <0.001 | 0.019 | 0.010 | 0.558 | 0.523 |
| MIF | 3.750097+0.0036191*(Age-5.429570703)-0.0572151*(sex-1)-0.2957733*DG2-0.1230528*DG3-0.1951975*DG4 |  | <0.001 | 0.036 | 0.461 | 0.020 | 0.426 |
| MIG CXCL9 | 2.766412-0.0080103*(Age-5.429570703)-0.0331037*(sex-1)-0.093874*DG2+0.5324967*DG3+0.0327453*DG4 |  | <0.001 | 0.352 | <0.001 | 0.582 | 0.521 |
| MIP1a CCL3 | 0.0840669-0.0076413*(Age-5.429570703)-0.0232925*(sex-1)-0.2391243*DG2-0.0502936*DG3-0.0126728*DG4 |  | 0.455 | 0.279 | 0.848 | 0.923 | 0.837 |
| MIP1b CCL4 | 1.817059-0.0133068*(Age-5.429570703)+0.0195456*(sex-1)-0.3826782*DG2+0.08482*DG3-0.0480604*DG4 |  | <0.001 | <0.001 | 0.356 | 0.293 | 0.621 |
| PDGF-BB | 2.574855-0.0002706*(Age/10^-2-3.39210287)-0.1434405*(sex-1)-0.1162082*DG2-0.0676393*DG3-0.1076164*DG4 |  | <0.001 | 0.458 | 0.714 | 0.222 | 0.073 |
| RANTES CCL5 | 3.556956-0.0142618*(Age/10^-1-1.841766237)-0.2461141*(Age/10^0.5-0.7368562073)-0.066119*(sex-1)+0.0157017*DG2-0.0240854*DG3-0.0176637*DG4 | <0.001 | <0.001 | 0.811 | 0.755 | 0.646 | 0.048 |
| SCF | 1.912271-0.0044246*(Age-5.429570703)-0.0282318*(sex-1)-0.2448652*DG2+0.1431278*DG3-0.0525531*DG4 |  | <0.001 | 0.006 | 0.175 | 0.315 | 0.533 |
| SCGFb | 4.358666+0.1422111*(Age/10^-0.5-1.357116884)-0.0132769*(sex-1)-0.4244375*DG2+0.3477771*DG3-0.1132401*DG4 |  | <0.001 | 0.001 | 0.017 | 0.113 | 0.828 |
| SDF-1α CXCL12 | 2.557649+0.0002718*(Age-5.429570703)-0.029705*(sex-1)-0.0032159*DG2+0.0458772*DG3+0.0064467*DG4 |  | <0.001 | 0.926 | 0.270 | 0.754 | 0.098 |
| TNF-α | 1.465943+0.3578067*(ln(Age/10)+0.6107250224)-1.163955*(Age/10^0.5-0.7368562073)-0.0449007*(sex-1)+0.015989*DG2-0.0791841*DG3+0.0405324*DG4 | <0.001 | <0.001 | 0.872 | 0.494 | 0.481 | 0.365 |
| TNF−β | -0.4436116-0.029722*(Age-5.429570703)-0.2592307*(sex-1)+0.2852874*DG2+1.230995*DG3+0.1250962*DG4 |  | 0.024 | 0.454 | 0.007 | 0.579 | 0.186 |
| TRAIL | 1.616795+0.003506*(Age-5.429570703)+0.0823782*(sex-1)+0.0999714*DG2+0.078111*DG3+0.0288589*DG4 |  | <0.001 | 0.355 | 0.544 | 0.651 | 0.138 |
| VCAM1 | 4.512854-0.3629383*(Age/10^0.5-0.7368562073)+0.3033317*(Age/10^0.5*ln(Age/10)+0.4500165237)+0.0034149*(sex-1)-0.2582278*DG2+0.1219749*DG3-0.1097954*DG4 | 0.002 | <0.001 | <0.001 | 0.079 | 0.002 | 0.908 |
| VEGF | 1.323723-0.0134596*(Age-5.429570703)-0.065366*(sex-1)+0.0166445*DG2-0.1912634*DG3+0.0357526*DG4 |  | <0.001 | 0.899 | 0.224 | 0.646 | 0.333 |
